# The Association Between Flexibility and Injury Incidence Among Female Collegiate Rhythmic Gymnasts

**DOI:** 10.7759/cureus.97926

**Published:** 2025-11-27

**Authors:** Saki Nakai, Ruriko Shibayama, Kumiko Kisara, Misuzu Hashizume, Yasuharu Nagano

**Affiliations:** 1 Graduate School, Japan Women’s College of Physical Education, Tokyo, JPN; 2 Faculty of Physical Education, Japan Women’s College of Physical Education, Tokyo, JPN

**Keywords:** ankle, foot, hip, lumbar spine/low back, survey

## Abstract

Aims/objectives

Flexibility is essential for rhythmic gymnastics performance, yet both insufficient and excessive flexibility may contribute to injury risk. However, the relationship between flexibility and injury in rhythmic gymnasts remains unclear. This study prospectively investigated the association between flexibility characteristics and injury occurrence in collegiate female rhythmic gymnasts.

Materials and methods

Forty-four collegiate rhythmic gymnasts were assessed for flexibility in side splits, forward bends, backbends, and straight leg raises. Over a 14-week period, athletes reported weekly whether physical complaints affected their training or performance using the Oslo Sports Trauma Research Center (OSTRC) questionnaire. Participants were categorized into two groups: those who reported one or more complaints at any point during the 14-week period and those who did not report any complaints. Flexibility differences between groups were analyzed using unpaired t-tests.

Results

The overall prevalence of complaints was 61.2%. Athletes with lower-back complaints demonstrated significantly reduced backbend flexibility (p < 0.01). Those with ankle complaints showed significantly greater backbend flexibility (p < 0.05) but significantly lower side-split flexibility (p < 0.05). Athletes with foot complaints showed significantly greater flexibility in the left straight leg raise (SLR) (p < 0.05). No significant flexibility differences were observed among athletes with hip or thigh complaints.

Conclusions

Both limited and excessive flexibility were associated with increased injury risk, depending on the anatomical region. Regular assessment of flexibility profiles may help guide individualized training and injury prevention strategies for rhythmic gymnasts.

## Introduction

Rhythmic gymnasts are prone to various musculoskeletal injuries, particularly in the lower back and lower extremities, including ankle sprains, metatarsal fractures, hip joint pain, and stress fractures [1-3]. A prospective study conducted in Norway [4] found that 20% of reported overuse injuries among rhythmic gymnasts were low back pain. Meanwhile, another study [5] showed that 80% of the athletes in the sample reported back pain. Injuries in rhythmic gymnastics tend to be chronic rather than acute in nature, necessitating longitudinal surveillance for accurate assessment.

Injuries among rhythmic gymnasts are often characterized by chronic rather than acute conditions [2,6-8]. Therefore, research on injuries in rhythmic gymnastics should primarily focus on chronic injuries. Previous studies investigating injuries in rhythmic gymnasts have employed various research methods [2,5,8]. When missed practices are used as an indicator of disability in surveys, there is a risk of underestimating the actual physical problems experienced by athletes.

Although rhythmic gymnastics requires a high degree of flexibility, it is conceivable that both excessive and insufficient flexibility could increase the risk of physical complaints or injury. However, the relationship between flexibility and injury in rhythmic gymnasts remains unclear. Unlike previous studies that defined injuries based on time loss or clinical diagnosis, the present study focuses on physical complaints reported by athletes, which may include overuse symptoms and subclinical conditions. Consequently, we conducted this study to assess the flexibility of rhythmic gymnasts and to prospectively examine the risk factors for injury, based on the definition of “any complaint of physical injuries” and using the Oslo Sports Trauma Research Center (OSTRC) questionnaire [9]. This validated tool enables longitudinal tracking of all symptomatic physical problems experienced by athletes, including those that do not lead to missed training sessions.

## Materials and methods

Participants

Forty-four female collegiate rhythmic gymnasts participated in this study (mean age: 19.0 ± 2.0 years; height: 1.60 ± 0.05 m; weight: 50.1 ± 4.6 kg). All participants were members of university teams competing at the top collegiate level in Japan, including both individual and group rhythmic gymnasts. They trained approximately six days per week, with each training session lasting about 300 minutes. The purpose and procedures of the study were explained in detail, both verbally and in writing, and informed consent was obtained from all participants. The study was approved by the Research Ethics Committee of the Japan Women’s College of Physical Education (Approval No. 2021-14).

Study design and injury surveillance

Participants completed a weekly injury survey using the Japanese version of the OSTRC questionnaire [10] (Appendix 1), administered via Google Forms (Google, Mountain View, CA, USA) on smartphones. The questionnaire asked whether any physical complaints had affected their training or performance during the past seven days. The primary study period lasted 14 weeks (from early May to late August 2022). The study period corresponded to the in-season phase, during which athletes participated in both official and unofficial competitions. Notably, official competitions were held in the first and final weeks of the 14-week observation period. Because the frequency of physical complaints tends to increase around competition periods due to injury occurrence and accumulated fatigue, this period was intentionally selected to examine the relationship between flexibility and physical complaints under realistic training and competition conditions.

Flexibility assessment

**Figure 1:**
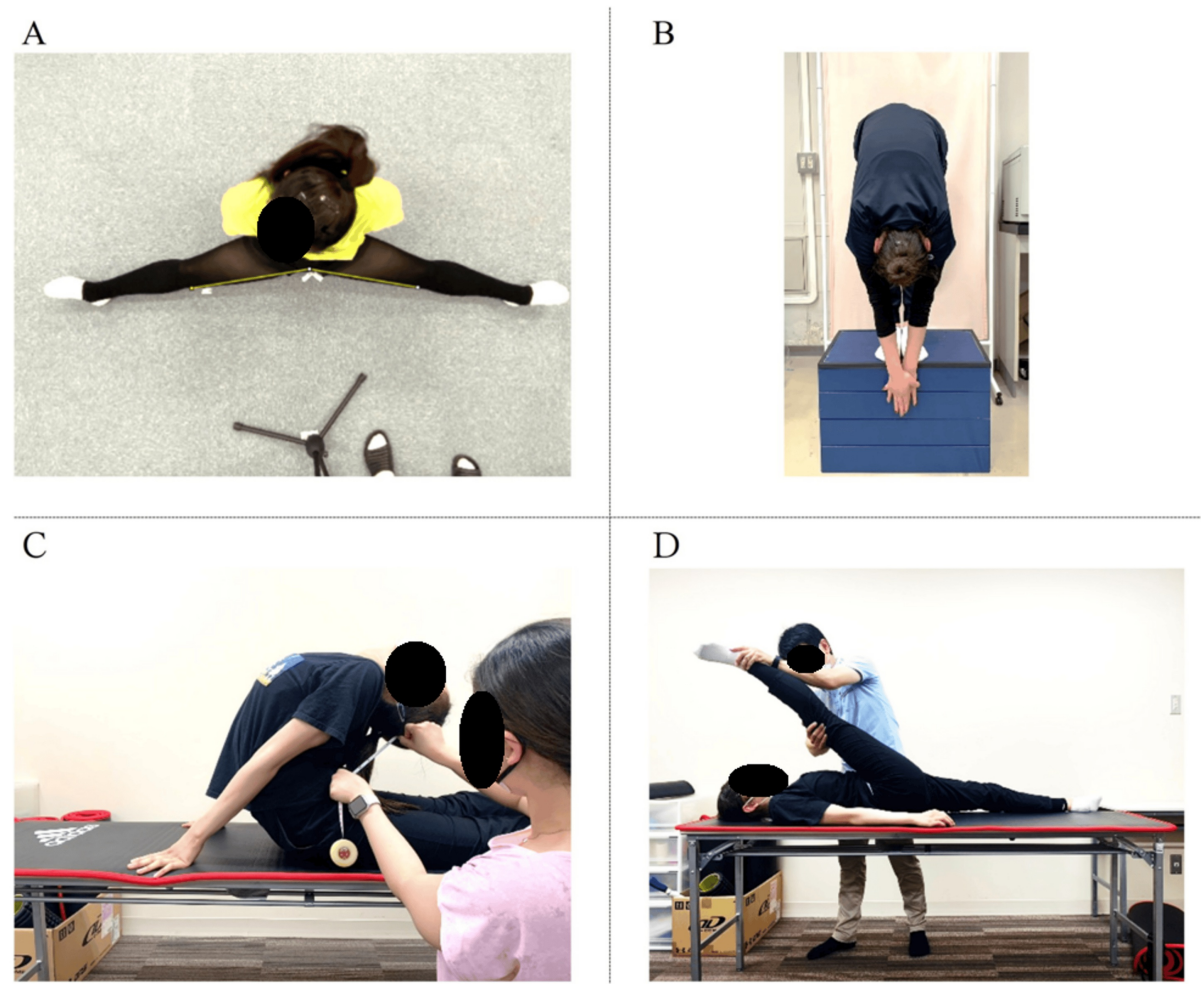
Flexibility assessment: (A) side split, (B) forward bend, (C) backbend, (D) straight leg raise (SLR)

Flexibility measurements were conducted during the pre-observation period, approximately one to two weeks before the start of the 14-week injury surveillance. Four flexibility tests were conducted (i.e., side split, forward bend, backbend, and straight leg raise (SLR)), as these represent the primary flexibility components required for rhythmic gymnastics performance (Figure 1).

Side Split

Overhead photographs were taken in the side-split position. Using ImageJ software (NIH, Bethesda, MD, USA), the angle formed by a line connecting the inner edge of each knee to the most indented point of the pelvis was calculated.

Forward Bend (Standing)

While standing on a measurement platform, participants bent forward with their middle fingers aligned. The distance between the fingertips and the platform surface (set at 0 cm) was measured.

Backbend

In a prone position with hands placed on the floor and elbows extended, participants bent backward. The distance between the buttocks and the head was measured.

Straight Leg Raise (SLR)

While supine, participants raised one leg with full knee extension. The angle between the raised leg and the floor was measured for both right and left sides.

All flexibility measurements were performed by an experienced physical therapist to ensure consistent measurement procedures. Two participants were excluded from statistical analysis due to pain experienced during the flexibility assessments.

Statistical analysis

The prevalence of physical complaints was calculated for each anatomical region by dividing the number of participants reporting complaints by the total number of respondents [11]. For classification, athletes who reported one or more physical complaints at any time during the 14-week period were categorized as the “complaint group,” while those who did not report any complaints were categorized as the “no-complaint group.” Multiple reports from the same athlete were counted as a single occurrence for grouping purposes. Flexibility was compared between these groups using unpaired t-tests. All statistical analyses were performed using IBM SPSS Statistics for Windows, Version 19.0 (Released 2010; IBM Corp., Armonk, NY, USA). The level of statistical significance was set at p < 0.05.

## Results

**Figure 2:**
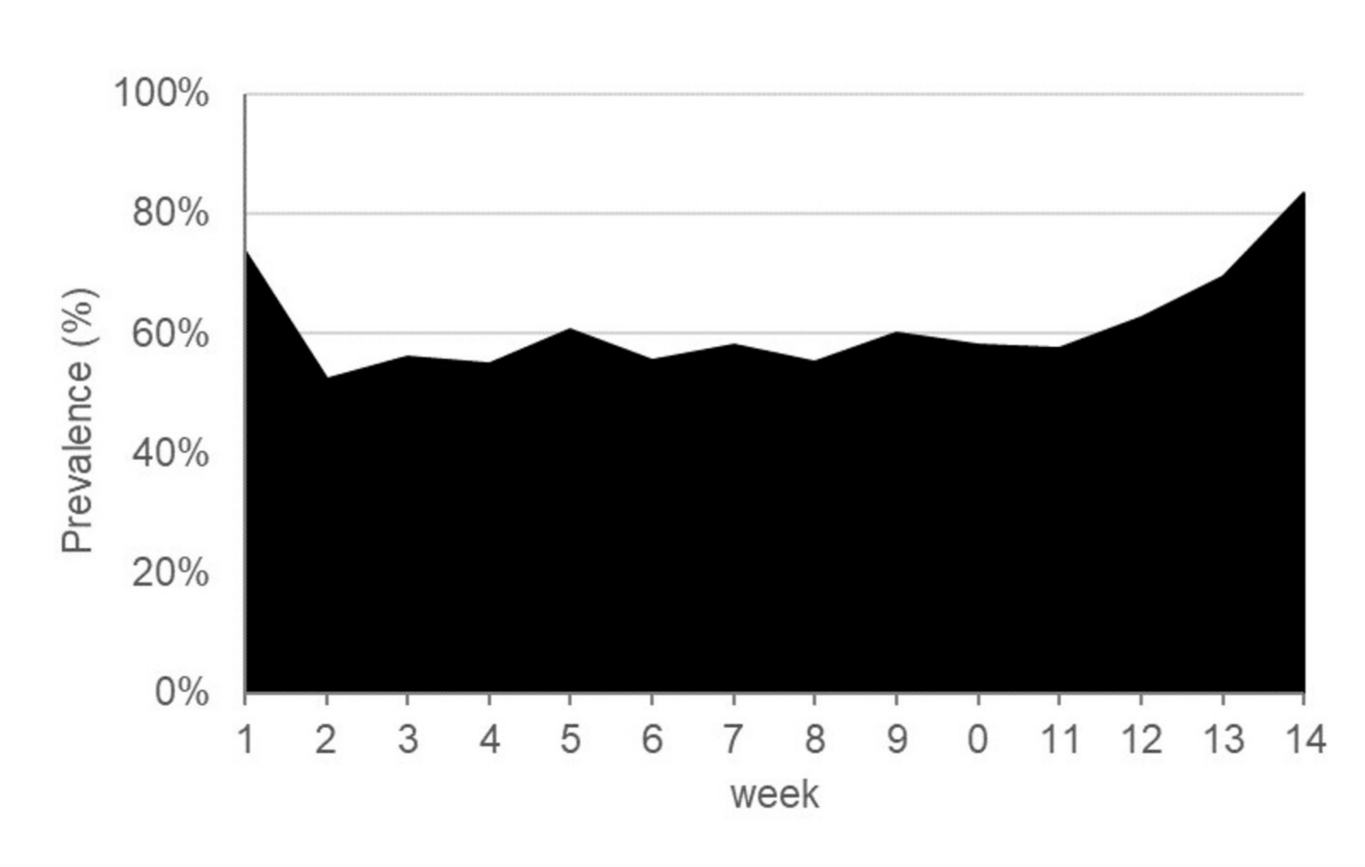
Temporal changes in the weekly prevalence of physical complaints among collegiate rhythmic gymnasts

A total of 532 weekly responses were collected during the 14-week study period, yielding a response rate of 90.5%. During the 14-week study period, the overall prevalence of physical complaints among participants was 61.2% (95% CI: 56.2-66.2). Among anatomical regions, the lower back exhibited the highest prevalence of complaints (42.8%, 95% CI: 39.3-46.3), followed by the ankle (19.8%, 95% CI: 17.2-22.4), foot (17.3%, 95% CI: 15.4-19.2), and hip/thigh (15.6%, 95% CI: 12.5-18.8). Temporal trends in overall complaint prevalence are presented in Figure 2. Table 1 summarizes the number and percentage of athletes who reported complaints in the four most frequently affected regions.

**Table 1:** Number and percentage of athletes reporting complaints in the most affected anatomical regions

Region	Number of athletes	Percentage (%)
Lower back	27	64.3
Ankle joint	19	45.2
Foot	13	31.0
Hip and thigh	14	33.3

Comparison of flexibility between groups

Flexibility measurements were compared between athletes who reported one or more complaints at any time during the 14-week period (complaint group) and those who did not report any complaints (no-complaint group) in each of the four most frequently affected anatomical regions.

Lower Back

Athletes with lower-back complaints demonstrated significantly reduced backbend flexibility compared with those without complaints (p < 0.01). On average, the complaint group showed approximately 8 cm less spinal extension range. No meaningful differences were observed between groups in forward bending, SLR, or side-split flexibility (Table 2).

**Table 2:** Flexibility comparison for lower-back complaints (mean ± SD) SLR: straight leg raise, n.s.: not significant.

	With lower back complaints	Without lower back complaints	p-value
Forward bending (cm)	26.6 ± 5.0	27.9 ± 5.1	n.s.
Backward bending (cm)	39.8 ± 6.5	31.9 ± 10.0	<0.01
SLR (right) (°)	112.0 ± 8.9	114.3 ± 7.3	n.s.
SLR (left) (°)	110.4 ± 7.6	112.3 ± 8.7	n.s.
Side split (°)	168.9 ± 8.0	169.9 ± 7.3	n.s.

Ankle Joint

Athletes who reported ankle complaints exhibited significantly greater backbend flexibility (p < 0.05) compared with those without complaints, with an average difference of about 5 cm. In contrast, they demonstrated significantly lower side-split flexibility (p < 0.05), with a reduction of approximately 6° compared with those without complaints. No other flexibility measures differed significantly between the two groups (Table 3).

**Table 3:** Flexibility comparison for ankle complaints (mean ± SD) SLR: straight leg raise, n.s.: not significant.

	With ankle complaints	Without ankle complaints	p-value
Forward bending (cm)	27.6 ± 5.1	26.7 ± 5.1	n.s.
Backward bending (cm)	34.1 ± 9.8	39.4 ± 7.0	<0.05
SLR (right) (°)	114.2 ± 9.0	111.7 ± 7.8	n.s.
SLR (left) (°)	113.4 ± 8.2	109.3 ± 7.3	n.s.
Side split (°)	166.1 ± 7.9	171.9 ± 6.5	<0.05

Foot

Athletes with foot complaints showed significantly greater flexibility in the left SLR test (p < 0.05) compared with those without complaints. The average difference between groups was about 6°. No significant differences were observed in backbend, side-split, or forward bend flexibility (Table 4).

**Table 4:** Flexibility comparison for foot complaints (mean ± SD) SLR: straight leg raise, n.s.: not significant.

	With foot complaints	Without foot complaints	p-value
Backward bending (cm)	36.4 ± 6.4	37.3 ± 9.6	n.s.
SLR (right) (°)	116.2 ± 8.2	111.4 ± 8.1	n.s.
SLR (left) (°)	115.3 ± 7.8	109.2 ± 7.3	<0.05
Side split (°)	172.4 ± 5.7	167.8 ± 8.1	n.s.

Hip and Thigh

No significant differences in flexibility were observed between athletes with and without hip or thigh complaints. The mean values for backbend, side-split, forward bending, and SLR were similar in both groups (Table 5).

**Table 5:** Flexibility comparison for hip/thigh complaints (mean ± SD) SLR: straight leg raise, n.s.: not significant.

	With hip complaints	Without hip complaints	p-value
Backward bending (cm)	36.9 ± 10.0	37.1 ± 8.2	n.s.
SLR (right) (°)	113.6 ± 10.1	112.5 ± 7.6	n.s.
SLR (left) (°)	112.1 ± 9.4	111 ± 7.2	n.s.
Side split (°)	171.9 ± 5.4	167.9 ± 8.4	n.s.

## Discussion

This study examined the relationship between flexibility and injury occurrence in collegiate rhythmic gymnasts using prospective injury surveillance over a 14-week period. The overall prevalence of physical complaints was 61.2%, indicating that a majority of athletes trained while experiencing symptoms. This prevalence is higher than the 37% reported in a previous study involving younger rhythmic gymnasts using the same OSTRC questionnaire [6], suggesting that Japanese collegiate gymnasts may be exposed to greater physical demands or are more likely to continue training despite experiencing physical complaints.

Lower back

Complaints in the lower back were the most frequently reported, consistent with previous studies [1,12]. Athletes who reported such complaints demonstrated significantly reduced flexibility in the backbend test at baseline measurement. Continuing training despite limited spinal extension may be a contributing factor to lumbar stress during the observation period. In rhythmic gymnastics, skills requiring extreme spinal extension are frequently performed [13]. When flexibility is insufficient, these athletes may be forced to move beyond their physiological range, potentially increasing mechanical load and the risk of injury. Targeted interventions to improve spinal extension flexibility through proper alignment and core control may be beneficial in reducing injury risk.

Ankle

In contrast to the findings for the lower back, athletes with ankle complaints demonstrated greater backbend flexibility. This may reflect a pattern of generalized joint hypermobility, a known risk factor for ankle laxity. Female athletes with generalized hypermobility may experience increased ligamentous laxity due to hormonal fluctuations, such as during ovulation, which has been linked to increased anterior talofibular ligament length [14]. Therefore, excessive spinal flexibility may be a marker of broader systemic laxity that predisposes gymnasts to ankle injuries.

Additionally, athletes with ankle complaints showed significantly reduced flexibility in the side-split test. Turnout in rhythmic gymnastics, similar to ballet, relies on hip external rotation. When this range of motion is insufficient, compensatory movement may occur at the ankle joint, increasing stress and potential for injury. Although some studies have not demonstrated a direct link between turnout and injury risk [15], the current findings suggest that hip mobility limitations may still influence ankle loading patterns.

Foot

Athletes with foot complaints exhibited significantly greater flexibility in the left straight leg raise test. The most commonly reported issue was plantar fasciitis. The superficial back line, a myofascial chain connecting the plantar fascia to the hamstrings [16], may play a role in this association. High hamstring flexibility may decrease the tension within this chain, reducing dynamic support of the foot during loading tasks and potentially contributing to overuse injuries such as plantar fasciitis.

Clinical implications

This study identified region-specific relationships between flexibility and injury occurrence among collegiate rhythmic gymnasts. These findings suggest that both limited and excessive flexibility may act as injury risk factors, depending on the anatomical region and movement demands. Coaches and medical professionals working with rhythmic gymnasts should implement regular flexibility assessments across key joints and tailor training programs accordingly [17]. For athletes with limited flexibility, improving mobility with appropriate alignment and motor control may be effective in reducing mechanical overload. For those with excessive flexibility, especially in hypermobile athletes, enhancing joint stability through neuromuscular control and strength training may help mitigate injury risk. As seen in the backbend results, while reduced flexibility was associated with complaints in certain areas, excessive flexibility was also linked to complaints in other areas. Integrating individualized flexibility profiles into injury prevention strategies could contribute to safer and more effective training in rhythmic gymnastics.

Limitations

This study has limitations that should be considered. The sample size was relatively small and limited to collegiate gymnasts, which may restrict generalizability. Flexibility measures were static, and dynamic flexibility or sport-specific movement patterns were not evaluated. Despite these limitations, the prospective design provides novel insights into the role of flexibility in rhythmic gymnastics injuries.

## Conclusions

This prospective study demonstrated that both insufficient and excessive flexibility were associated with region-specific injuries in collegiate rhythmic gymnasts. Reduced backbend flexibility was linked to lower back complaints, while excessive backward bend flexibility and limited side split were associated with ankle complaints. Greater hamstring flexibility was related to foot complaints. These findings highlight the importance of individualized flexibility assessments in rhythmic gymnastics. Incorporating flexibility profiles into screening and training programs may assist coaches and clinicians in developing targeted prevention strategies to reduce injury risk and support safe athletic participation.

## Appendices

Appendix 1

**Table 6:** Japanese version of the Oslo Sports Trauma Research Center (OSTRC) Overuse Injury Questionnaire. Adapted from: [10] (Creative Commons Attribution 4.0 International (CC BY 4.0)).

Question	Response Options
Q1. Have you had any difficulties participating in normal training and competition due to physical problems during the past week?	□ Full participation without physical problems □ Full participation, but with physical problems □ Reduced participation due to physical problems □ Cannot participate due to physical problems
Q2. To what extent have you reduced your training volume due to physical problems during the past week?	□ No reduction □ To a minor extent □ To a moderate extent □ To a major extent □ Cannot participate at all
Q3. To what extent have physical problems affected your performance during the past week?	□ No effect □ To a minor extent □ To a moderate extent □ To a major extent □ Cannot participate at all
Q4. To what extent have you experienced pain related to your sport during the past week?	□ No pain □ Mild pain □ Moderate pain □ Severe pain
Q5. If you have a physical problem, please select the most complained anatomical area from below.	□ shoulder □ elbow and upper arm □ wrist and forearm □ finger □ low back □ pelvis and glute □ hip and thigh □ knee □ shank □ ankle □ foot □ internal medical problem □ other
Q6. Did the problem in this week occur as a new problem or continue from before?	□ Yes, it sustained new. □ No, it have continued previously.
Q7. If you have other physical problems, please select the anatomical areas from below.	□ shoulder □ elbow and upper arm □ wrist and forearm □ finger □ low back □ pelvis and glute □ hip and thigh □ knee □ shank □ ankle □ foot □ internal medical problem □ other
